# Short-stem prostheses in primary total hip arthroplasty: A meta-analysis of randomized controlled trials

**DOI:** 10.1097/MD.0000000000005215

**Published:** 2016-10-28

**Authors:** Shao-Chuan Huo, Fan Wang, Lu-Jue Dong, Wei Wei, Jing-Qi Zeng, Hong-Xing Huang, Qing-Min Han, Rui-Qi Duan

**Affiliations:** a Guangzhou University of Chinese Medicine, Baiyun District; b Department of Orthopedics, Guangzhou University of Chinese Medicine Third Affiliated Hospital, Guangzhou University of Chinese Medicine, Liwan District, Guangzhou City, Guangdong Province, China.

**Keywords:** meta-analysis, randomized controlled trials, short-stem prostheses, total hip arthroplasty

## Abstract

**Background:**

Short-stem (SS) prostheses require less resection of the femoral neck, produce a more physiological load pattern in the proximal femur, reduce stress shielding, and aid bone conservation and are, therefore, beneficial for young patients. Conventional cementless implants in total hip arthroplasty (THA) have shown excellent clinical results; however, it is unclear whether SS prostheses can obtain the same clinical and radiological outcomes. We conducted a meta-analysis of randomized controlled trials (RCTs) to evaluate whether SS prostheses are superior to conventional implants after primary THA.

**Methods:**

We reviewed the literature published up to June 2016 from PubMed, Web of Science, and the Cochrane Library to find relevant RCTs comparing SSs and conventional stems in primary THA. Quality assessment was performed by 2 independent reviewers. The RevMan 5.3 software program of the Cochrane Collaboration was used to analyze the data. Random- or fixed-effect models were used to calculate standardized mean differences (SMDs) and 95% confidence intervals (CIs) for each comparison.

**Results:**

Six RCTs involving 552 patients with 572 hips were identified. Strong evidence indicated that SS prostheses were more effective for reducing thigh pain than conventional implants (*I* ^2^ = 46%, *P* = 0.002; risk ratio [RR], 95% CI 0.15, 0.04–0.49). However, there were no significant differences between the 2 groups in Harris Hip Scores (*I* ^2^ = 0%, *P* = 0.84; SMD, 95% CI 0.02, −0.15–0.18), Western Ontario and McMaster Universities Osteoarthritis Index Scores (*I* ^2^ = 0%, *P* = 0.35; SMD, 95% CI 0.09, −0.10–0.27), femoral offset of stem (*I* ^2^ = 0%, *P* = 0.57; SMD, 95% CI 0.06, −0.16–0.29), and leg-length discrepancy (*I* ^2^ = 79%, *P* = 0.88; SMD, 95% CI 0.04, −0.44–0.51).

**Conclusion:**

SS prostheses achieve the same clinical and radiological outcomes as conventional implants, and were superior in terms of reducing thigh pain. But whether the postoperative thigh pain applied in 2nd-generation cementless prosthesis still needs further large-scale multicenter studies with longer follow-up to confirm.

## 1 Introduction

Total hip arthroplasty (THA) is a treatment for various hip diseases, such as osteonecrosis of the femoral head, development dysplasia hip, and hip arthritis. In primary THA, 2 types of prostheses are available: conventional stems (CSs) and short stems (SSs). CSs are a standard length of ∼150 mm, compared with SSs,^[1]^ which are <120 mm in length.^[^2 3^]^ Although excellent survival rates have been reported with conventional femoral stems in THA,^[^4 5^]^ proximal stress shielding and thigh pain often occur after THA.^[3]^ In young patients who have potential revision surgery, it is necessary to conserve bone mass and extend the service life of prostheses. SSs have the characteristics of preserving bone, preventing stress shielding, and providing favorable conditions for revision, which are advantages for young patients. These SSs focus on metaphyseal fixation. Reimeringer et al,^[6]^ in an analysis of a finite element model, demonstrated that reducing stem length to <105 mm was not associated with the stability of implants. SS designs depend upon a stable metaphyseal fit and require optimal proximal load transfer, and are beneficial to conserve bone mass and reduce thigh pain. The uncemented metaphyseal-engaging SSs exhibit excellent outcomes in clinical and radiography studies, as they conserve stability and enable proximal bone remodeling closer to the metaphysis than CSs at 5-year follow-up.^[7]^ Santori and Santori^[8]^ have confirmed that a short proximal loading femoral component showed satisfactory results in patients of a mean age of 51 years at 8-year follow-up, suggesting that the absence of a diaphyseal portion of the stem did not influence the stability of the prosthesis.

Although a number of randomized controlled trials (RCTs) have compared the effectiveness of SS versus CS in primary THA,^[^9 10^]^ no meta-analysis has compared the clinical and radiographic outcomes between the 2 stems. Therefore, we conducted a meta-analysis of the RCTs available in the literature to evaluate the effectiveness of short versus conventional femoral stems in primary THA.

## 2 Materials and methods

### 2.1 Ethics statement and guidelines

The meta-analysis of the RCTs comparing the effectiveness of SS versus CS in primary THA involved no animal experiments or direct human trials, and neither a special ethics review nor ethical approval was therefore necessary. Our study was conducted according to the preferred reporting items for systematic reviews and meta-analyses statement.^[11]^

### 2.2 Inclusion and exclusion criteria

Inclusion criteria were as follows: prospective, randomized, controlled study designs; patients with hip diseases, such as osteoarthritis, osteonecrosis, traumatic arthritis, or femoral neck fracture, who were scheduled to undergo primary THA; comparing the clinical and radiographic outcomes of SS versus CS in primary THA; studies involving the Harris Hip Scores (HHS), the Western Ontario and McMaster Universities Osteoarthritis Index (WOMAC) scores, femoral offset, leg-length discrepancy, and thigh pain; the age of the patients was not restricted, and the minimum average follow-up time was 6 weeks; and the language of the publications was limited to English. Exclusion criteria were as follows: patients who had undergone primary THA with cemented implants; and noncontrolled clinical trials.

### 2.3 Information sources and search

We carried out a systematic electronic search in PubMed, Cochrane Library, and Web of Science from inception to June 2016. We combined relevant keywords to build the search strategy, including total hip arthroplasty, total hip replacement, short stem, conventional stem, and standard stem. The search terms were ((((total hip arthroplasty) OR total hip replacement)) AND (((short stem) OR conventional stem) OR standard stem)). We checked all potential references with NotExpress (Sun Yat-Sen University, Guangdong, China) and removed the duplicates.

### 2.4 Data extraction

Two investigators extracted the relevant data independently from each study as follows: details of participants, interventions, and outcomes. Two reviewers first screened the potentially relevant literature on the basis of titles and abstracts, and 2 investigators independently determined each eligible published study based on full text according to inclusion criteria. Disagreements between 2 investigators were arbitrated by another 2 investigators, and unpublished studies were not searched. Means, standard deviations, sample sizes, and adverse events were extracted from primary outcome measures. In the absence of standard deviations, we obtained them from *P* values. An assumption that the same standard deviations came from outcome measures could be used in the SS and CS groups. First, we obtained a *t* value from a *P* value, then we obtained the standard error from the *t* value, and finally calculated a standard deviation from the standard error. We pooled the data and produced graphs with the Review Manager (RevMan 5.2) software program.

### 2.5 Quality appraisal

The quality of evidence and strength of recommendations were rated by the GRADE system,^[12]^ which offered 2 grades (strong and weak) of recommendations. However, there were some other factors that affected the quality of the recommendations, which were defined as high quality, moderate quality, low quality, and very low quality. We used the Cochrane Collaboration tool to assess risk of bias in randomized trials.^[13]^ The metaAnalyst software program was used to analyze the sensitivity of the individual trials. The preferred reporting items for systematic reviews and meta-analyses statement was used as a basis for reporting the randomized trials.^[11]^

### 2.6 Statistical analysis

Statistical heterogeneity of the pooled data was examined using the *I* ^2^ statistic. It is defined that an *I* ^2^ of less than 40% is low, 30% to 60% is moderate, 50% to 90% is substantial, and 75% to 100% is considerable.^[14]^ The overlapping ranges represented the arbitrary and uncertain acknowledgment. If heterogeneity was lower than 50%, we used the fixed-effects model. If *I* ^2^ was higher than 50%, we considered that there was statistical heterogeneity, and random-effects models were used to conduct predefined sensitivity analyses. Egger test was used to assess publication bias, these tests are based on the assumption that the accuracy of effect quantity increases with sample size. Subgroup analysis was conducted by HHS, WOMAC, femoral offset, and leg-length discrepancy. We used GRANDE guidelines^[15]^ to deal with dichotomous outcomes, and risk ratios (RRs) and 95% confidence intervals (CIs) were calculated. Standardized mean differences (SMDs) or RR and 95% CI were used to pool estimates of each analysis. A *P* value of less than 0.05 was statistically significant.

## 3 Results

### 3.1 Study selection

**Figure 1:**
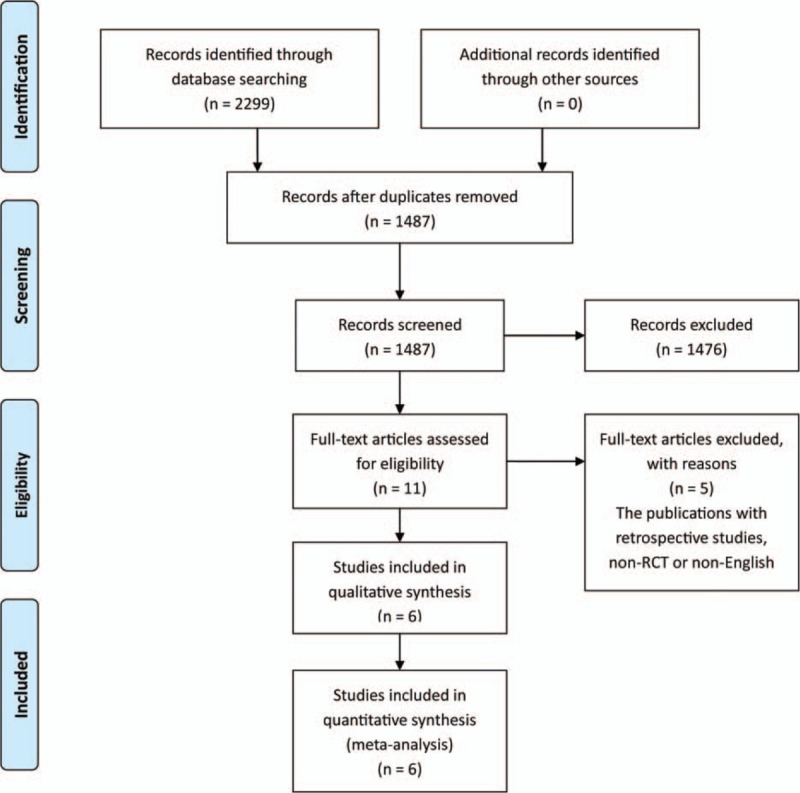
Flow chart of study selection.

Our electronic search yielded 2299 articles, of which, 812 duplicate articles were removed and 1476 articles were excluded based on the titles and abstracts. The remaining 11 articles were retrieved for full-text review, and 5 articles were excluded because they were retrospective, non-RCT, or non-English language studies. Finally, 6 articles were prospectively randomized controlled studies, which were deemed eligible for inclusion. Figure 1 summarizes the process of identifying eligible studies.

### 3.2 Study characteristics

Table 1 shows the characteristics of the 6 RCTs included in our meta-analysis. All but 1 of the 6 studies included patients with osteoarthritis, osteonecrosis, traumatic arthritis, and femoral neck fracture.^[16]^ Of the 6 included studies, 3 used posterolateral approaches,^[^9 10 17^]^ 2 used a direct lateral approach,^[^18 19^]^ and 1 used a minimally invasive anterolateral approach.^[16]^ There was adequate random sequence generation in 2 studies,^[^9 17^]^ but 4 articles did not report it.^[^10 16 18 19^]^ The studies included a total of 265 patients using SSs, with a total of 287 patients using CSs. The individual sample sizes of the studies ranged from 43 to 140 patients. Anteroposterior and lateral radiographs of each hip in 2 planes were performed at preoperative diagnosis and postoperative follow-up. The measurement of femoral offset and leg-length discrepancy was based on these images. Four studies reported that weight bearing on the affected limb was allowed as comfort permitted,^[^9 10 16 18^]^ 1 reported immediate full weight-bearing after surgery using 2 crutches,^[19]^ but 1 did not describe this point.^[17]^ Thigh pain was scored based on a 10-point visual analog scale in 2 studies,^[^9 17^]^ but another study reported thigh pain graded as none, mild, moderate, or severe.^[10]^ Although the measurement was different in the different studies, we could establish whether thigh pain occurred. The age of patients in the 6 studies was a mean of 62.6 years (range 51.8–76.0 years). The follow-up period of the studies varied, but the mean follow-up time was 2.2 years (range 0.115–4.8 years).

**Table 1:**
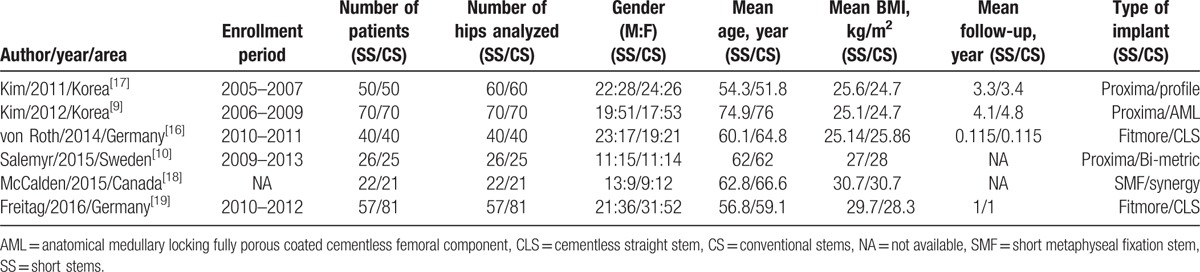
Characteristics of the included studies.

### 3.3 Risk of bias

**Figure 2:**
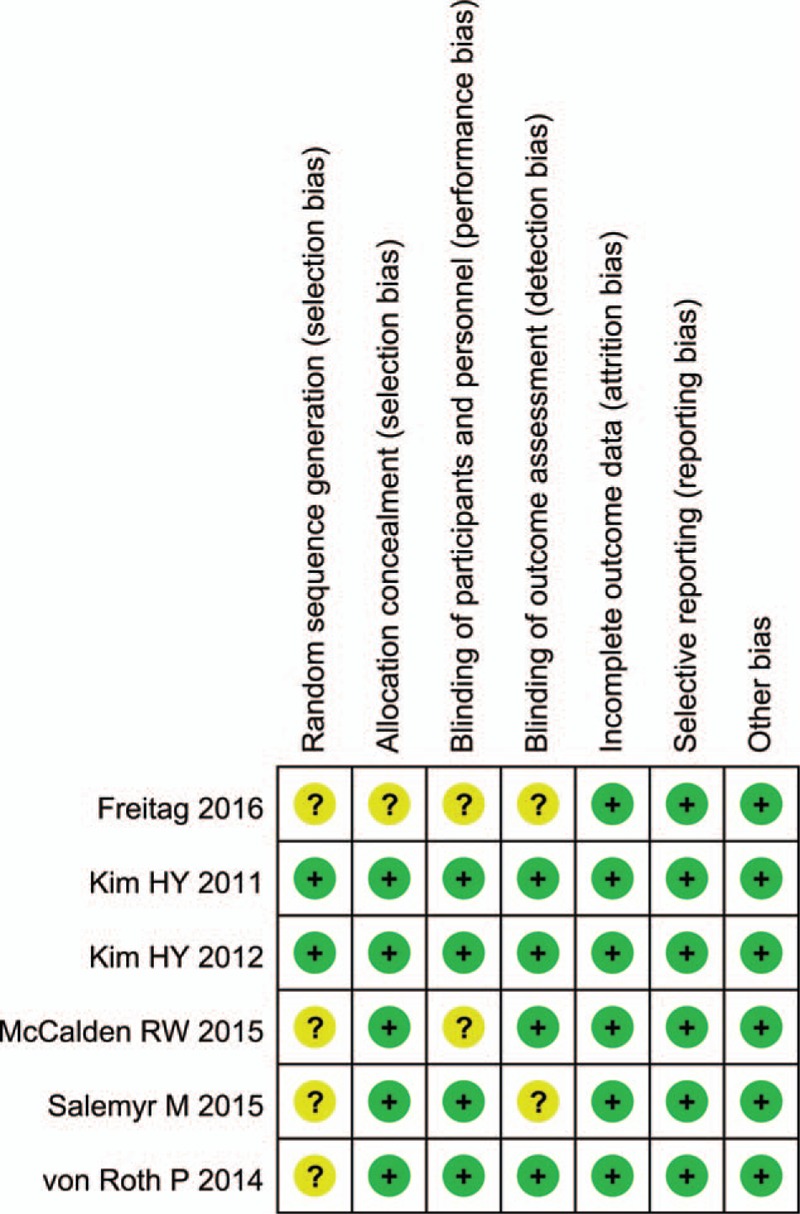
Risk of bias abstract: review authors’ judgements about each risk of bias item for each included study.

**Figure 3:**
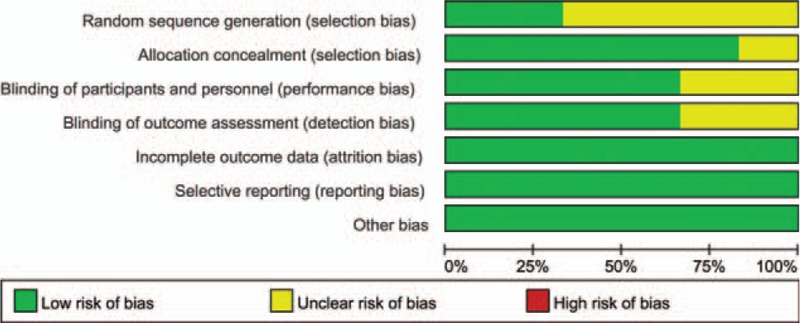
Risk of bias graph: review authors’ judgements about each risk of bias item presented as percentages across all included studies.

Figure 2 summarizes the assessment of risk of bias for individual trials. In general, the 6 trials were judged as having a low risk of bias. Participants and personnel were blinded in 4 studies, while 2 studies did not describe this process clearly. The blinding of outcome assessment was performed in 4 studies, whereas 2 studies did not report it. Incomplete outcome data were regarded as low risk of bias in the 6 studies. Because 4 studies reported the reason of patient loss, and the rate of patient loss was less than 10%. Although there was no patient loss in the other 2 studies. Based on the life-style or privacy which did not influence the measurement of clinical outcomes, selective reporting was regarded as low risk of bias in the 6 RCTs. All prostheses were supplied by companies, but patients randomly received either SSs or CSs on the basis of a sequential numbering system, and we considered that the other sources of domain bias carried a low risk of bias. Figures 2 and 3 summarize the detailed risk of bias related to the methodological quality of the 6 studies. Table 2 represents the quality of evidence and strength of recommendation according to Grades of Recommendations Assessment, Development and Evaluation profiler.

**Table 2:**
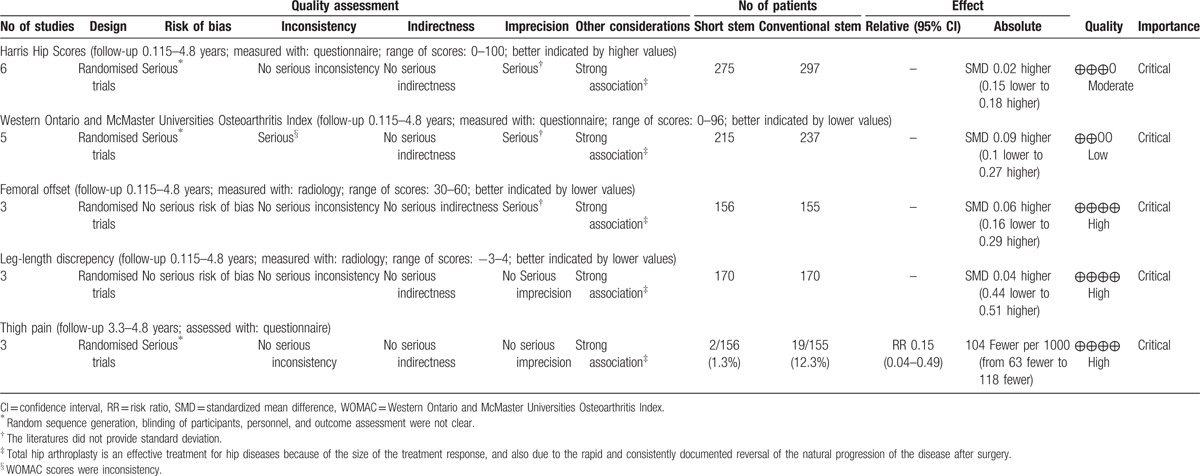
The quality of evidence and strength of recommendations was based on the GRADE system.

### 3.4 Meta-analysis results

**Figure 4:**
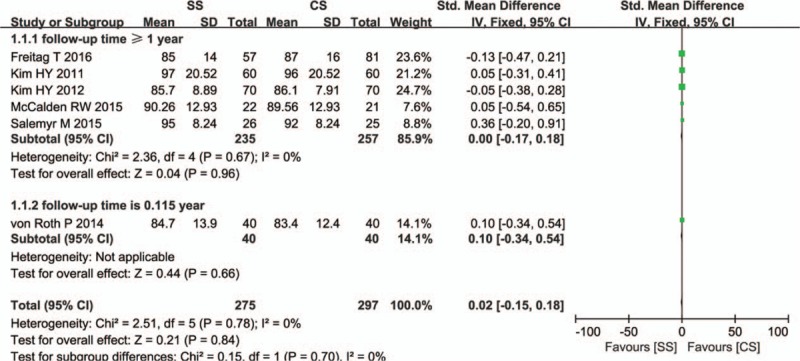
Comparison of HHS between short stems and conventional stems in primary THA. HHS = the Harris Hip Score, THA = total hip arthroplasty.

Six studies including 552 patients reported the HHS. The combination of data from the studies revealed that there was no significant difference between the 2 stems in mean HHS at the latest follow-up (SMD = 0.02, 95% CI, −0.15–0.18; *P* = 0.84). There was no statistical heterogeneity in the pooled data (*I* ^2^ = 0%). Publication bias was tested by Egger test and showed no change in significance (*P* = 0.577). Sensitivity analysis by sequential omission of individual studies did not change the result significantly (*P* = 0.774). Subgroup meta-analyses showed no significant differences at the end of follow-up (*P* = 0.70; Fig. 4).

**Figure 5:**
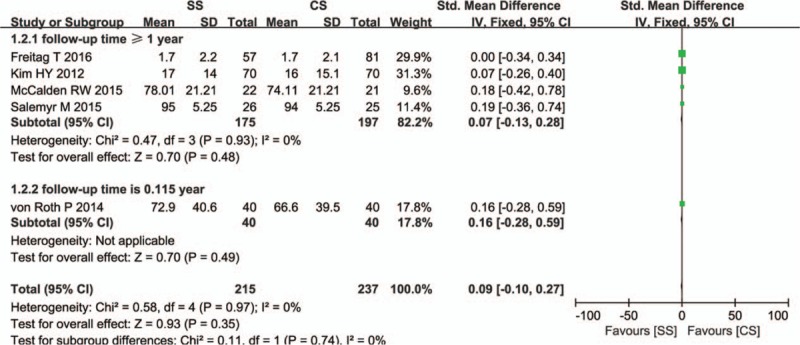
Comparison of WOMAC scores between short stems and conventional stems in primary THA. THA = total hip arthroplasty, WOMAC = Western Ontario and McMaster Universities Osteoarthritis Index.

WOMAC score was adopted in 5 studies. All the studies demonstrated no significant difference between SS and CS (SMD = 0.09, 95% CI, −0.10–0.27; *P* = 0.35). There was no statistical heterogeneity between the individual studies (*I* ^2^ = 0%). Publication bias was tested by Egger test and showed a significant change (*P* = 0.004). Sensitivity analysis by sequential omission of individual studies did not change the result significantly (*P* = 0.962). Our meta-analysis found that there were no significant differences between the subgroups (*P* = 0.74; Fig. 5).

**Figure 6:**
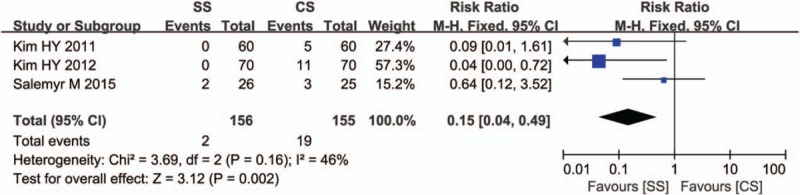
Comparison of thigh pain between short stems and conventional stems in primary total hip arthroplasty (THA).

Three studies reported thigh pain, including 2 of 146 patients in the SS group and 19 of 145 patients in the CS group. We found statistically significant differences in thigh pain between the 2 groups (RR = 0.15, 95% CI, 0.04–0.49; *P* = 0.002). Heterogeneity among the studies was moderate (*I* ^2^ = 46%). Egger test to assess potential publication bias showed no change in significance (*P* = 0.153). Sensitivity analysis showed a significant change by sequential omission of individual studies (*P* = 0.001; Fig. 6).

**Figure 7:**
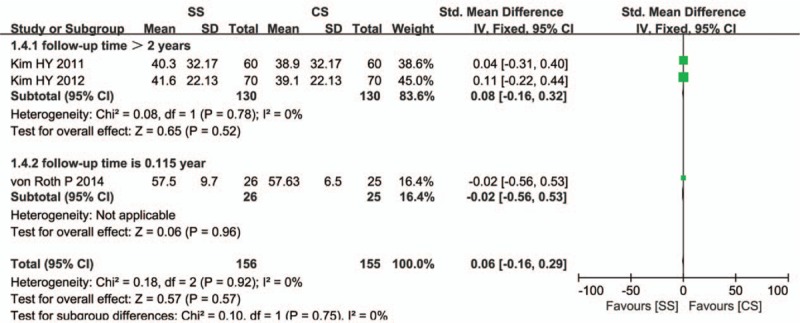
Comparison of femoral offset between short stems and conventional stems in primary total hip arthroplasty (THA).

Three studies including 320 patients reported femoral offset of stems. Pooled analysis demonstrated that there was no statistically significant difference in femoral offset between the SS and CS groups (SMD = 0.06, 95% CI: −0.16–0.29, *P* = 0.57). There was no statistical heterogeneity of the pooled data (*I* ^2^ = 0%). Egger test to assess potential publication bias showed no change in significance (*P* = 0.502). Sensitivity analysis showed no significant change by sequential omission of individual studies (*P* = 0.911). Our meta-analysis found that there were no significant differences between the subgroups (*P* = 0.75; Fig. 7).

**Figure 8:**
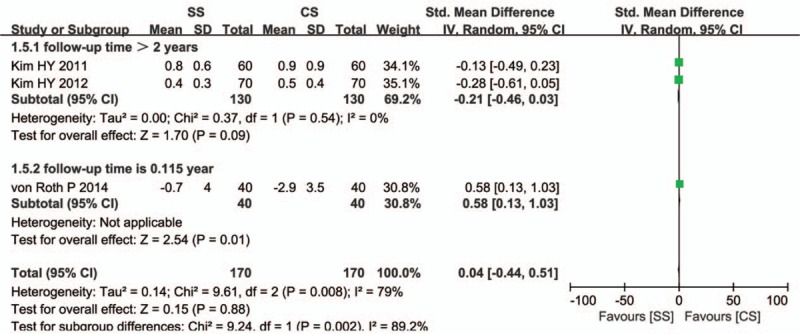
Comparison of leg-length discrepancy between short stems and conventional stems in primary total hip arthroplasty (THA).

Leg-length discrepancy data were available in 3 studies, and the meta-analysis showed no significant difference in leg-length discrepancy between SS and CS groups (SMD = 0.04, 95% CI: −0.44–0.51, *P* = 0.88). However, heterogeneity existed among individual trials (*I* ^2^ = 79%); therefore, we used the random-effects model. Egger test to assess potential publication bias showed no change in significance (*P* = 0.333). There was a significant difference between the subgroups regarding leg-length discrepancy (*P* = 0.002, *I* ^2^ = 89.2%). Sensitivity analysis showed significant change by sequential omission of individual studies (*P* = 0.008; Fig. 8).

## 4 Discussion

This meta-analysis of 5 RCTs including 552 patients comparing the efficacy of SS and CS showed that SSs were superior to CSs in reducing thigh pain. We found no significant differences in HHS, WOMAC, femoral offset, and leg-length discrepancy between the 2 stems in primary THA.

HHS was examined in these 6 studies^[^9 10 16–19^]^ and WOMAC in 5 studies.^[^9 10 16 18 19^]^ The postoperative self-reported measures (HHS, WOMAC) were greatly improved comparing to the preoperative ones in the 2 designs of stems. Our meta-analysis found strong evidence indicated no difference in HHS and WOMAC when comparing SSs to CSs after THA. From our included studies, we found that the short follow-up time (6 weeks) did not influence the heterogeneity of the pooled results of HHS and WOMAC. We speculated that patients could obtain excellent clinical scores postoperatively in the early stage whatever prostheses were used. Cinotti et al^[20]^ reported that the HHS averaged 43 points (range 19–50) preoperatively and 88 points (range 73–100) at a minimum follow-up of 9 years, and WOMAC increased from 47 points (range 35–56) before surgery to 76 points at the final follow-up. Patel et al^[21]^ reported an average HHS of 88 for a cohort of ≥70 years and 93 for a cohort of ≤70 years at a minimum follow-up of 24 months (mean, 35 months; range, 24–60 months). Therefore, age and duration of follow-up may influence HHS and WOMAC. From Fig. 5, we found that the evaluation methods were not inconsistent in WOMAC,^[^9 19^]^ so the random-effects models were used, and the Egger test showed a significant change (*P* = 0.004).

Of the 3 studies in our analysis that reported thigh pain, our results indicate that SSs can reduce the incidence of postoperative thigh pain. Thigh pain after THA may be caused by micromotion of the stem^[^22 23^]^ or stem design.^[^24 25^]^ A pooled analysis of the data showed a low incidence of thigh pain in SSs when comparing with CSs, and several studies have demonstrated the low incidence of thigh pain with SSs,^[^21 26–28^]^ which can be attributed to the design of the femoral stem. Shin et al^[29]^ also reported that a lower incidence of thigh pain in SSs than in CSs. An SS can reduce proximal stress shielding through diaphyseal fixation of the femoral stem and the development of excellent mechanical transmission. Kolisek et al^[30]^ reported that the rate of thigh pain with 2nd-generation uncemented stems was less than 1%, but there were some other literatures reported the rates were up to 2%.^[^31 32^]^ So, further large-scale multicenter studies are required to confirm the rates of postoperative thigh pain using modern cementless stems. The authors reviewed some published literatures about cementless total hip replacement with 2nd-generation components in recent years,^[^31 33^]^ and found cortical hypertrophy of the distal femoral stems may be the site of stress shielding and did not cause thigh pain, it was an adaptive bone remodeling response to mechanical stress. Obviously, the postoperative thigh pain is not necessarily to be applied in 2nd-generation cementless prosthesis.

Our meta-analysis found that there were no significant differences in the presence of femoral offset and leg-length discrepancy after primary THA using SSs versus CSs. There was no statistical heterogeneity in femoral offset between the 2 groups in 3 studies (Fig. 7); 2 studies clearly described that the perpendicular distance was from the neutral long axis of the femur to the center of the femoral head,^[^16 17^]^ but 1 study did not describe this measurement.^[9]^ Significant heterogeneity was seen in the measurement of leg-length discrepancy in the trials (Fig. 8). One trial measured the difference of leg length as the distance between the inferior margin of both ischial tuberosities (interischial line) and the top of the lesser trochanter of each hip comparing the operated and the nonoperated side;^[17]^ a positive value represents a longer and a negative value represents a shorter limb length. Another trial measured the difference in leg length as the distance between the acetabular teardrop figures and the lesser trochanter.^[16]^ One trial did not describe the measurement.^[9]^ The differences in measurement method may influence the heterogeneity of the pooled results. But several studies have also shown no significant differences in leg-length discrepancy between SSs and CSs after THA.^[^9 16 17 34^]^

Our meta-analysis has some limitations. First, heterogeneities existed in the length of follow-up and prosthesis design among the RCTs in our study. Second, only 6 RCTs with 552 patients were included, non-English language papers were not included, and unpublished studies were not identified; thus, some related studies may have been missed and some publication bias may exist in our analysis. Third, the detailed outcomes such as migration of femoral components, infection of prostheses, femoral bone remodeling, and radiolucent line or loosening around implants were not assessed and require future study. Owing to the absence of revision events, the reason for revision could not be assessed. Further large-scale multicenter studies with longer follow-up are required to confirm the findings.

## 5 Conclusion

SS prostheses achieve the same clinical and radiological outcomes as conventional implants, and were superior in terms of reducing thigh pain. But whether the postoperative thigh pain applied in 2nd-generation cementless prosthesis still needs further large-scale multicenter studies with longer follow-up to confirm.

## Acknowledgments

The authors thank all the anonymous reviewers and editors for their helpful suggestions on the quality improvement of our paper.
